# Descriptive epidemiology of poliomyelitis cases due to wild poliovirus type 1 and wild poliovirus type 3 in Nigeria, 2000-2020

**DOI:** 10.11604/pamj.supp.2023.45.2.38079

**Published:** 2023-07-14

**Authors:** Philip Bammeke, Usman Said Adamu, Omotayo Bolu, Ndadilnasiya Waziri, Tesfaye Erberto, Aron Aregay, Peter Nsubuga, Eric Wiesen, Faisal Shuaib

**Affiliations:** 1 Centers for Disease Control and Prevention, Atlanta, Georgia, United States,; 2 National Primary Healthcare Development Agency, Abuja, Nigeria,; 3 African Field Epidemiology Network, Abuja, Nigeria,; 4 World Health Organization, Abuja, Nigeria,; 5 World Health Organization, Kyiv, Ukraine,; 6 Global Public Health Solutions, Atlanta, Georgia, United States

**Keywords:** Polio eradication, poliomyelitis, wild poliovirus, emergency operations center, supplementary immunization, vaccination campaign, poliovirus vaccine, acute flaccid paralysis

## Abstract

**Introduction:**

in August 2020, the World Health Organization African Region was certified free of wild poliovirus (WPV) when Nigeria became the last African country to interrupt wild poliovirus transmission. The National Polio Emergency Operations Center instituted in 2012 to coordinate and manage Nigerian polio eradication efforts reviewed the epidemiology of WPV cases during 2000-2020 to document lessons learned.

**Methods:**

we analyzed reported WPV cases by serotype based on age, oral poliovirus vaccine immunization history, month and year of reported cases, and annual geographic distribution based on incidence rates at the Local Government Area level. The observed trends of cases were related to major events and the poliovirus vaccines used during mass vaccination campaigns within the analysis period.

**Results:**

a total of 3,579 WPV type 1 and 1,548 WPV type 3 laboratory-confirmed cases were reported with onset during 2000-2020. The highest WPV incidence rates per 100,000 population in Local Government Areas were 19.4, 12.0, and 11.3, all in 2006. Wild poliovirus cases were reported each year during 2000-2014; the endemic transmission went undetected throughout 2015 until the last cases in 2016. Ten events/milestones were highlighted, including insurgency in the northeast which led to a setback in 2016 with four cases from children previously trapped in security-compromised areas.

**Conclusion:**

Nigeria interrupted WPV transmission despite the challenges faced because of the emergency management approach, implementation of mass vaccination campaigns, the commitment of the government agencies, support from global polio partners, and special strategies deployed to conduct vaccination and surveillance in the security-compromised areas.

## Introduction

The global declaration of smallpox eradication in 1979 was a catalyst that intensified the optimism that other human diseases could be eradicated globally with concerted effort. With the progress in the Americas after 1980, poliomyelitis (polio) caused by poliovirus infection was deemed among the next likely [1,2]. Polioviruses cause an acute enteric infection that uncommonly (<1% of infections) clinically manifests as acute flaccid paralysis (AFP) and can lead to death [3]. Paralytic polio is caused by one of three serotypes of wild poliovirus (WPV), type 1 (WPV1), type 2 (WPV2), and type 3 (WPV3), each with its unique epidemiology, case-to-infection ratio, immune response, and vaccine characteristics [3]. Liberating the world from indigenous WPV will be a significant global public health success after the eradication of smallpox (and for global animal health, rinderpest, a disease of cattle) and will further highlight the role of vaccines in eradicating viral diseases [4]. In 1988, the World Health Assembly (WHA) formally endorsed efforts to eradicate polio through the Global Polio Eradication Initiative (GPEI) by 2000 [5].

Global eradication of polio by the year 2000 was a challenging goal [6]. The efforts made by the GPEI after the 1988 declaration led to an enormous decline in paralytic polio cases from an estimated 350,000 cases in 125 countries in 1988 to <1,000 confirmed cases in 10 endemic countries by 2001 [7,8]. The last case of WPV2 detected was in 1999 in India, while the last case of WPV3 detected was in 2012 in Nigeria [3]. The eradication of WPV2 and WPV3 was certified in 2015 and 2019, respectively [3,9]. Countries still endemic for WPV1 transmission in 2016 were Afghanistan, Nigeria, and Pakistan [6]. With its latest identified WPV1 isolation in September 2016, Nigeria became the last country in the World Health Organization (WHO) Region of Africa (AFR) to interrupt WPV transmission with no detection of a paralytic case caused by WPV infection in 3 years in line with GPEI standard. Consequently, the Africa Regional Commission for the Certification of Poliomyelitis Eradication certified the Region indigenous WPV-free on August 25, 2020 [10].

Up to 2016, insecurity, low routine (essential) childhood immunization coverage, and low national resource commitments were major obstacles to polio eradication efforts in many countries, including the Nigerian polio eradication program [11]. The challenges were each predominant at various points, and they affected the implementation of GPEI strategies, especially Supplementary Immunization Activities (SIAs) and AFP surveillance, including case investigation and collection of stool specimens. The issue of insecurity, particularly in insurgent-held areas, became more challenging to the Nigerian polio program in the 2010s [12,13]. In 2012, the Nigerian government established the National Polio Emergency Operations Centre (NEOC) as an operational arm within the National Primary Health Care Development Agency (NPHCDA) to coordinate and manage all polio eradication activities [14,15]. The NEOC enabled direct coordination and collaboration by the Government with several GPEI partners to advance polio eradication efforts. Similar coordination structures were later set up in seven priority states. Following the interruption of WPV in Nigeria, the NEOC conducted a review study on the epidemiology of WPV cases in Nigeria from 2000-2020. The main objective was to characterize the WPV cases and corroborate the impact of key polio eradication activities and events on the WPV trend in Nigeria.

## Methods

**Study design:** this was a cross-sectional study that reviewed WPV cases in Nigeria during 2000-2020. Spatio-temporal analysis of the cases was combined with a qualitative assessment of polio eradication activities to describe the WPV epidemiology.

**Setting:** the study setting is Nigeria, a West-African country that extends into the Atlantic Ocean and in the northeast shares Lake Chad borders with Cameroon, Chad, and Niger. The review of the WPV cases was across the country´s 36 states plus the Federal Capital Territory (FCT), and the 774 Local Government Areas (LGA) across the 36 states and the FCT.

**Study population:** the WPV cases were based on the AFP surveillance that targeted children under 15 years; circulating vaccine-derived poliovirus (cVDPV) cases were not included in this study. Children under 5 years of age were routinely targeted for SIAs, i.e. mass vaccination campaigns used to administer poliovirus vaccines to a large population within a short period.

**Variables:** these included the annual number of WPV1 and WPV3 cases; the age, gender, and Oral Polio Vaccine (OPV) immunization dose history of the cases, LGA-level population estimates, WPV incidence rates, the key/critical events in the Nigerian polio eradication journey, and the poliovirus vaccines used during polio SIAs. Only the poliovirus vaccines administered towards the interruption of WPV during SIAs conducted as National Immunization Plus Days (NIPDs) or Sub-national Immunization Plus Days (SIPDs) in selected states, and WPV outbreak responses were included.

**Data sources:** the NEOC provided the data on WPV cases per year which included demographic data, the critical Nigerian polio eradication events, the number of polio SIAs conducted per year, and the poliovirus vaccines each year. The LGA-level population estimates were based on the 2006 Nigerian census data.

**Bias and control:** the varying population over time and differences in population across the LGAs required the use of incidence rates to map WPV cases reported each year. The key events/milestones verifiable in the literature were included.

**Study size:** nationwide AFP surveillance is the gold standard for detecting cases of poliomyelitis, and the incidence rate was based on the GPEI AFP surveillance sensitivity indicator of at least one case of non-polio AFP detected annually per 100,000 population aged less than 15 years. In endemic regions, the rate is two per 100,000 to ensure even higher sensitivity [16]. Nigeria even increased to three per 100,000 at some point between 2016-2017 [12].

**Data analysis:** we conducted a descriptive statistical analysis of the cases by gender, age group, and OPV doses immunization history using SPSS statistical software version 23. Considering the target for SIAs is children under 5 years and for AFP surveillance is children under 15 years, the ages of the WPV cases were grouped into 0-11 months, 12-23, 24-35, 36-47, 48-59, >59 months, and unknown. The immunization history of total OPV doses based on the recall by parents or caregivers for each case as documented in the database of the laboratory-confirmed WPV cases were grouped based on the standard of analysis used in Nigeria into 0, 1-3, 4-7, >7, and unknown. The epidemic curve was plotted based on the count of WPV1 and WPV3 cases per year in stacked bars. Spatio-temporal analysis was done, and choropleth maps were produced to show the annual geographic distribution of the WPV incidence rates in affected LGAs using the Geographic Information System (GIS) software, ArcGIS. The incidence rates were determined by dividing the number of WPV cases in each affected LGA by the projected total population based on the 2006 Nigerian census population figures, which were adjusted backward and forward in time using an estimated growth rate of 2.7%. The mapping classification for the incidence rates per 100,000 population based on the sensitivity of AFP surveillance was categorized into five groups of 0.1-1.0, 1.1-2.0, 2.1-3.0, 3.1-5.0, and >5.0.

The events/milestones that directly/indirectly advanced polio eradication efforts in Nigeria were classified as positive, and those that directly/indirectly weakened the efforts were negative. The events were plotted against the annual number of WPV cases. The number of SIAs conducted each year was tabulated with the type of poliovirus vaccines administered and the number of WPV1 and WPV3 cases per year. Qualitative inferences were made across the various results to holistically describe the epidemiology of WPV in Nigeria and assess the impact of the highlighted events/milestones on the observed trends. Biomedical literature was used to support the interpretation of observed findings.

**Ethical approval:** no personally identifiable information associated with the WPV1 or WPV3 cases in Nigeria was used in the study. This analysis of surveillance and investigation data was considered non-research public health practice.

## Results

**Incidence of WPV in Nigeria, 2000-2020:** a total of 5,127 laboratory-confirmed WPV cases were reported with onset during 2000-2020 of which 3,579 (69.8%) of the paralytic cases were caused by WPV type 1 with onset during 2000-2016, and 1,548 (30.2%) of the paralytic cases were caused by WPV type 3 with onset during 2000-2012.

**Figure 1:**
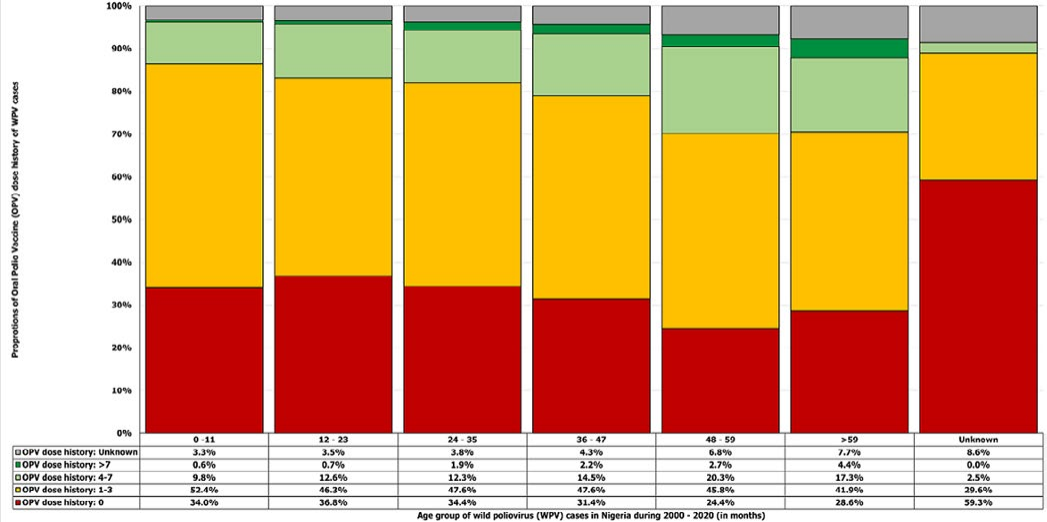
caretaker recall total oral polio vaccine dose history (routine and supplementary immunization) at the time of initial investigation of acute flaccid paralysis in children with confirmed wild poliovirus cases by age group, Nigeria 2000-2016

**Age and gender of persons with wild poliovirus:** of the 5,127 laboratory-confirmed WPV cases, the ages of 74 persons with cases that had an OPV dose history are unknown, while both the ages and OPV dose history of 7 persons with cases are unknown. The median age of children with WPV cases was 24 months; the youngest was in the first month of life (0 months), and the oldest person with a confirmed case was 204 months (17 years). The proportion of males was 56.1%, and females, 43.9%. The age range with the largest proportion of cases was 12-23 months at 34.4%, followed by 24-35 months at 32.4% (Table 1). The parental recall OPV dose history of the largest proportion of children with WPV cases was zero at 34.3% (Table 2). Of the zero-dose cases, the age group with the largest proportion of cases was 12-23 months at 36.8%, followed by 24-35 months at 34.4% (Figure 1).

**Table 1:** distribution by gender and age group of persons with wild poliovirus cases, Nigeria, 2000-2016

Characteristic	Frequency (n=5,127)	Percent
**Gender**		
Male	2,877	56.1
Female	2,250	43.9
**Age range (months)**		
0 - 11	338	6.6
12 - 23	1,766	34.4
24 - 35	1,663	32.4
36 - 47	736	14.4
48 - 59	295	5.8
>59	248	4.8
**No age data**	81	1.6

**Table 2:** caretaker-recall oral poliovirus vaccine immunization dose history of children with wild poliovirus cases at the time of investigation of acute flaccid paralysis, Nigeria, 2000-2016

Oral polio vaccine dose history of wild poliovirus cases	Frequency (n=5,127)	Percent
0	1,759	34.3
1	842	16.4
2	879	17.1
3	678	13.2
4	373	7.3
5	189	3.7
6	73	1.4
7	38	0.7
8	31	0.6
9	5	0.1
10	25	0.5
>10	20	0.4
**No data**	215	4.2

**Figure 2:**
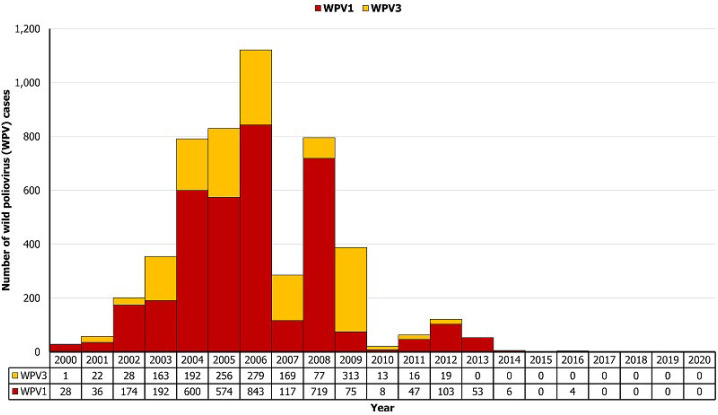
number of confirmed annual wild poliovirus type 1 and type 3 cases, Nigeria, 2000-2020

**Annual trend of the wild poliovirus cases:** at least one WPV case was reported each year during 2000-2016, except in 2015, and the onset of paralysis of the last confirmed case was in August 2016 (Figure 2). There was an increase in WPV cases from 2000 to 2006, with the peak period during 2004-2006. The largest number of WPV cases in a calendar year was in 2006 with 1,122 cases. The pattern alternated significantly during 2006-2009 (including vacillation of WPV1 and WPV3 incidence), with a 95% reduction in 2010 to 21 WPV cases from 388 in 2009 but increased again to 62 in 2011 and 122 in 2012. The WPV curve flattened in Nigeria in 2012, with 53 cases in 2013 and 6 in 2014; the last WPV3 isolation was in November 2012, and WPV1 from a case-contact of a positive index case with a date of collection in September 2016 which was later than the August 2016 onset of the last confirmed case.

**Figure 3:**
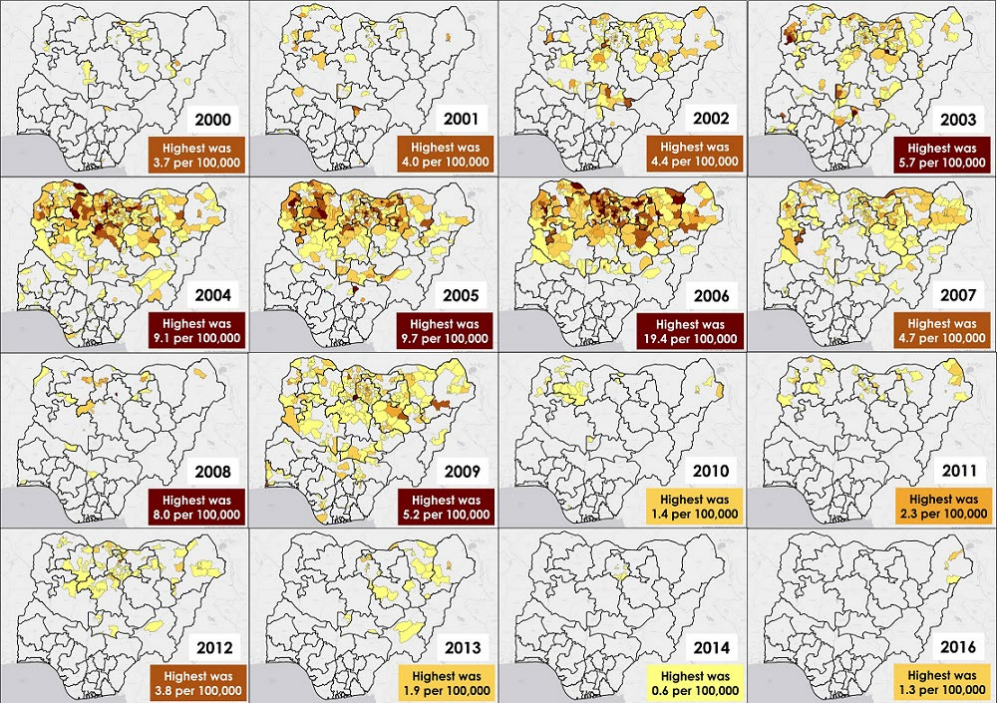
annual wild poliovirus incidence rates at the Local Government Area level in Nigeria, 2000-2016

**Geospatial distribution of the wild poliovirus cases:** the yearly geographic distribution maps of the spatio-temporal analysis in Figure 3 showed that wild poliovirus (WPV1 + WPV3) was detected and reported from multiple LGAs in states over the analysis period. Each of the six geographic zones in Nigeria (north-central, north-east, north-west, south-east, south-south, and south-west) were affected at some point with at least one LGA having a case. Most of the high-incidence LGAs were in the northern part of the country every year and the high incidence rates were observed across the northern axis from the north-central zone to the north-eastern and north-western zones, especially during 2002-2009. The highest annual incidence rate of wild poliovirus per 100,000 population observed from a single LGA was 19.4 in 2006 in a state in the north-west zone. All the incidence rates of >10.0 per 100,000 over the analysis period were in 2006 across six LGAs, all in the north-west zone. After the year 2006, 2005 was the year that had the LGA with the next highest incidence rate of 9.7 per 100,000 population from the north-west zone, and then 9.1 per 1000,000 population in 2004 in an LGA also from the north-west zone. Wild poliovirus 1 was last detected in Borno state in the northeast in 2016; three LGAs were affected.

**Figure 4:**
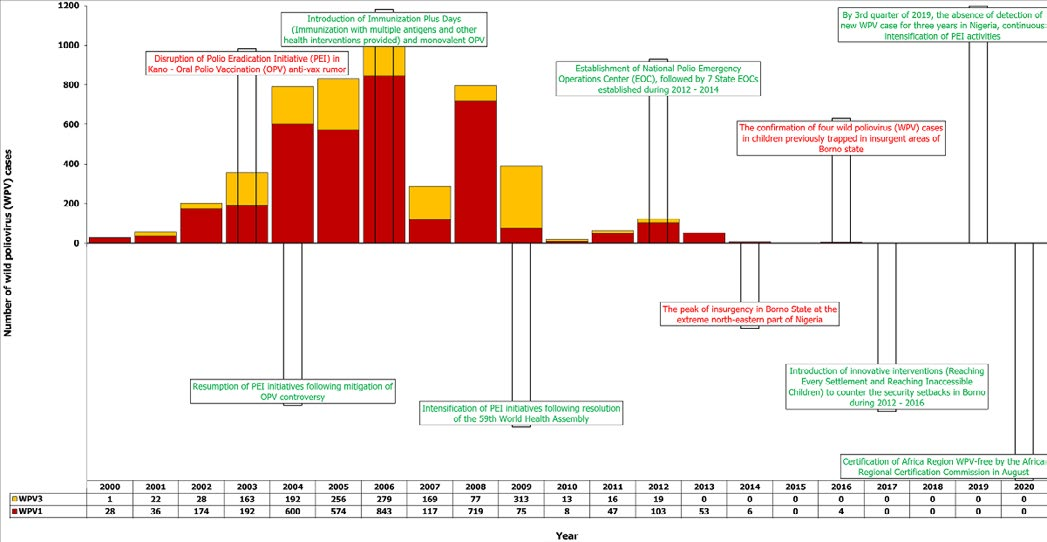
timeline of historic events in the Nigerian polio eradication journey and wild poliovirus cases each year from 2000-2020

**Major events/milestones during the period:** ten key events/milestones were highligted and annotated by year of occurrence and were plotted against the annual number of WPV cases. Three of these events negatively affected polio eradication in Nigeria. In contrast, seven of these had positive effects directly or indirectly, including the 2020 Regional Certification milestone resulting from the other positive events (Figure 4). The key events/milestones are: 1) disruption of PEI in Kano State due to OPV anti-vaccination rumor in 2003, with banning of campaigns there and in some other northern states (negative event); 2) resumption of PEI initiatives following mitigation of OPV controversy in 2004 (positive event); 3) introduction of Immunization Plus Days (IPDs) (immunization with multiple antigens and other health interventions provided) and monovalent OPV in 2006 (positive event); 4) intensification of PEI following the resolution of the 59th WHA in 2009 (positive event); 5) the establishment of NEOC in 2012, followed by the seven State Emergency Operation Centers established during 2012- 2014 (positive event); 6) the peak of insurgency in Borno State in the north-east around Lake Chad limited the reach of PEI activities, including SIAs, routine vaccination, and surveillance, in the security-compromised areas of the state from 2014 (negative event); 7) The confirmation of four WPV cases in children previously trapped in insurgent areas of Borno state in 2016 (negative event); 8) Introduction of innovative interventions (reaching every settlement, reaching inaccessible children to counteract the security setbacks in Borno during 2012-2016 (positive event); 9) By the 3rd quarter of 2019, the absence of detection of new wild poliovirus cases for three years in Nigeria, with the continuous intensification of PEI activities (positive milestone); 10) Certification of Africa Region polio-free by the Africa Regional Certification Commission in August 2020 (positive milestone).

**Administered poliovirus vaccines during the period:** five types of poliovirus vaccines were used to vaccinate eligible children across all the implemented polio SIAs within the analysis period (Table 3). Trivalent oral poliovirus vaccine (tOPV), which contains the three types of Sabin strain polioviruses, was the vaccine in use as of 2000 and last used in 2016. In 2006 which was the peak year of WPV cases, monovalent oral poliovirus vaccine type 1 (mOPV1) was introduced, and monovalent oral poliovirus vaccine type 3 (mOPV3) was introduced in 2007 and both were last used in 2011. The bivalent oral poliovirus vaccine (bOPV), which contains only type 1 and type 3 Sabin strain polioviruses, was introduced in 2010. Inactivated poliovirus vaccine (IPV) administered through intramuscular or subcutaneous route (0.5ml) was introduced in 2014 to induce/boost individual immunity against all polioviruses, while the fractional inactivated poliovirus vaccine (fIPV), which is 0.10ml of IPV given by intradermal administration, was first used in 2016. By the time AFR was declared WPV- free in August 2020, only bOPV and IPV/fIPV were in use specifically for WPV-preventive SIAs in Nigeria.

**Table 3:** number of poliovirus vaccine supplementary immunization activities conducted as national or sub-national immunization plus days by year and vaccine formulation used, and number of wild poliovirus cases by type, Nigeria, Aug 2000 -2020

Year	Number of SIA rounds^†^ each polio vaccine was used each year - National [Sub national - selected states]					No. of cases	
	tOPV	mOPV1	mOPV3	bOPV	IPV/fIPV	WPV1	WPV3
2000	2 [2]	-	-	-	-	28	1
2001	4 [1]	-	-	-	-	36	22
2002	3 [4]	-	-	-	-	174	28
2003	2 [7]	-	-	-	-	192	163
*2004	3 [1]	-	-	-	-	600	192
2005	4 [3]	-	-	-	-	574	256
2006	2 [1]	0 [3]	-	-	-	843	279
2007	1 [2]	1 [3]	0 [3]	-	-	117	169
2008		1 [4]	1 [2]	-	-	719	77
2009	1 [1]	2 [4]	1 [1]	-	-	75	313
**2010	2 [1]	0 [1]	0 [3]	2 [5]	-	8	13
***2011	2 [4]	0 [1]	1 [0]	2 [7]	-	47	16
2012	1 [0]	-	-	1 [6]	-	103	19
2013	1 [1]	-	-	1 [7]	-	53	0
2014	1 [3]	-	-	3 [7]	0 [5]	6	0
2015	2 [2]	-	-	1 [4]	0 [3]	0	0
2016	2 [1]	-	-	0 [6]	0 [2]	4	0
2017	-	-	-	2 [4]		0	0
2018	-	-	-	2 [2]	0 [1]	0	0
2019	-	-	-	1 [3]	0 [1]	0	0
****2020	-	-	-	1 [0]	0 [2]	0	0
**Total**	33 [36]	4 [16]	3 [9]	16 [51]	0 [14]	3,579	1,548

† Polio SIA round (NIPD or SIPD) each poliovirus vaccine (oral and inactivated) was used in at least one state; even if a poliovirus vaccine type was used in some selected states, while another type was used in the other states during a round; *2004 - All states except Kano and Zamfara states, participated in February NIPDs; all except only Kano in March; **2010 - All states participated in January 2010 NIPDs, except Plateau; while all states participated in Nov 2010 NIDPs/Maternal, Newborn and Child Health Week, except Borno and Gombe; ***2011 - All states participated in January NIPDs, except Cross River and Oyo; while all states participated in February NIPDs, except Anambra, Enugu, and Oyo; ****2020 - Polio SIA rounds from January to August 2020 when the African region was certified free of WPV.

SIA - Supplementary immunization activities; NIPDs - National immunization plus days; SIPDs - Sub-National immunization plus days; tOPV - Trivalent oral poliovirus vaccine, containing Sabin strain types 1, 2 and 3; mOPV1 - Monovalent oral poliovirus vaccine Sabin type 1; mOPV3 - Monovalent oral poliovirus vaccine Sabin type 3; bOPV - Bivalent oral poliovirus vaccine, containing Sabin strain types 1 and 3; IPV - Inactivated poliovirus vaccine (0.5ml intramuscularly); fIPV - Fractional dose inactivated poliovirus vaccine (0.1ml intradermally); WPV1 - Wild poliovirus type 1; WPV3 - Wild poliovirus type 3.

## Discussion

It took Nigeria 20 years after the initial global target of polio eradication to reach the milestone when the African Region was certified WPV-free [6,10]. Anti-vaccination rumors adversely impacted the uptake of vaccines and disruption of polio SIAs in 2003 and after; rumors spread emanated from northern state political and religious leaders, primarily in Kano, the most populous state in the northern axis of the country [17]. The state authorities in four northern states with high populations of targeted children suspended polio SIAs for some of the rounds during 2003-2004 statewide or in some cases certain parts of a state, resulting in decreased acceptance of OPV in the northern parts of Nigeria [18]. It was not surprising that the peak period of WPV cases in Nigeria was subsequently from 2004-2006 and mainly in the northern regions, as community trust in the vaccine and the PEI was not quickly regained. Wild poliovirus was endemic in northern Nigeria because of chronically low coverage achieved in the routine immunization (RI) program and SIAs [19]. Even though most of the cases were from the northern axis of the country, each of the six geographical regions from the north to the south of Nigeria had at least an affected LGA at some point over the analysis period. Afghanistan, India, Pakistan, and Nigeria were the remaining polio-endemic countries by 2005. Wild poliovirus exportations majorly from northern Nigeria and northern India subsequently caused several outbreaks and paralyzed >1500 children in previously polio-free African and Asian countries (i.e. >1 year after the last confirmed indigenous WPV case) [20,21]. The experience in Nigeria is also unique in that there was the discovery of a resurgence of endemic WPV transmission detected in 2016 after undetected transmission for the two prior years, due to the insurgency beginning in 2012 and peaking in around 2014 that limited both immunization and surveillance activities [22,23].

Oral Polio Vaccine (OPV) is inexpensive, easy to administer by trained lay personnel, and after a number of dose exposures, provides good protection against poliomyelitis and poliovirus infections [19]. With the GPEI introduction of mOPV1 in 2006 (and mOPV3 in 2007), mOPV1 was used predominantly for SIAs to preferentially target WPV 1; this had a substantial impact on WPV 1 transmission but did not interrupt circulation [5]. Since WPV type 2 had been last detected globally in 1999, and apparent effectiveness per dose against WPV 1 was higher for mOPV1 and bOPV than tOPV because of type 2 poliovirus interference with type 1 seroconversion with the first doses, GPEI switched the focus to bOPV as the preferred SIAs vaccine in WPV-affected countries in 2010 [5]. By 2014, the risk from continued regular use of tOPV that contained Sabin strain type 2 was considered to outweigh the benefits [24]. It was critical that all countries using tOPV needed to withdraw all tOPV in a synchronized manner within a short time frame during April 17-May 1, 2016, to avoid creating type 2 circulating vaccine-derived polioviruses (cVDPV2) [25,26]. The complete switch to bOPV from tOPV was done per the GPEI guidelines and synchronized globally by May 2016 [24]. There were substantial limitations in high-risk areas in surveillance, prior SIAs and RI coverage with tOPV before the switch, and the effectiveness of response Sabin OPV type 2 SIAs to outbreaks of cVDPV2 after the switch. These limitations have had complex ramifications for ongoing outbreaks as of 2022, including in Nigeria.

Despite all national and international commitments and efforts towards the end of the 2000s, Nigeria was still a polio-endemic country, along with Afghanistan, India, and Pakistan. The WHA declared that the completion of polio eradication was a programmatic emergency for global public health in 2012 [5]. This contributed to the creation of the Emergency Operations Center (EOC) structures in Nigeria. The EOC management approach at national and stated levels has been credited as one of the critical success factors in the Nigerian polio eradication program [15,26]. The EOC structure in Borno, with the support of the NEOC, was able to conduct the required outbreak responses effectively, following the detection of ongoing endemic transmission with confirmation of cases in children trapped in security-compromised areas [27]. The Reaching Every Settlement (RES) and Reaching Inaccessible Children (RIC) special interventions were implemented, wherein with the support of security personnel, eligible children trapped in or evacuated from security-compromised areas were vaccinated with OPV and enhanced AFP surveillance activities were conducted, aided by enhanced community informant reporting [12,28,29]. Satellite imagery was used to estimate unreached populations in inhabited settlements and the reach to these settlements over time with the special interventions were verified within a Vaccinator Tracking System [28,30].

Low childhood RI coverage in countries and areas allows poliovirus transmission to persist, or large outbreaks to occur when poliovirus is reintroduced [31]. With the administration of effective SIAs, many eligible children are vaccinated quickly with each round. In Nigeria, this substantially boosted the population and herd immunity against poliovirus [32]. Supplementary Immunization Activities cannot directly address underlying problems in a country´s RI program [33]. Nonetheless, Nigerian PEI had several capacities and GPEI-funded investments that were leveraged to strengthen RI systems and improve coverage and equity, such as OPV SIAs becoming “Immunization Plus Days” to deliver integrated health packages that included other RI vaccines and other health interventions (e.g., albendazole deworming) [15]. Sustaining polio eradication efforts in Nigeria, in turn, requires continuous improvement of RI service delivery [34]. From the perspective of this review, a strong RI program is crucial, considering most WPV cases in Nigeria over the analysis period had a caretaker recall immunization history of zero OPV doses.

Limitations: while the factors highlighted in this study substantially contributed to the observed pattern of WPV cases in Nigeria within the analysis period, there may have been other contributing and inhibiting factors specific to some areas. A similar review conducted at the state level may reveal specific factors that could have contributed to the interruption of WPV transmission peculiar to some states. Another limitation was that poliovirus/AFP surveillance was very suboptimal in 2000 and gradually strengthened, to be highly sensitive by 2010 except in the areas held by the insurgency during 2012-2016. Eradication of polio in its entire form is not limited to interrupting transmission of WPV but eradicating all types of clinical polio caused by polioviruses, i.e. polio due to vaccine-derived polioviruses (VDPVs) that emerge after prolonged circulation of Sabin poliovirus in under-immunized populations, and vaccine-associated paralytic poliomyelitis (VAPP) that can rarely occur in susceptible vaccine recipients or their susceptible close contacts following Sabin OPV [35]. The Nigerian polio program is currently challenged with an increasing number of cVDPV2 cases and outbreaks, well after the certification for the interruption of WPV transmission in August 2020.

## Conclusion

The interruption of WPV transmission in Nigeria with no case detected since 2016 was the outcome of several factors: 1) the commitment of the Nigerian government to polio eradication and support of the global community through the GPEI and other partners; 2) the establishment of the EOC structures for better coordination and management of all polio eradication efforts, with enhanced state and LGA ownership; 3) improved implementation of appropriate Supplementary Immunization Activity rounds and review of the types of poliovirus vaccines used; 4) strengthened and supervised AFP surveillance over the years; 5) special innovative immunization and surveillance activities in security-compromised areas; 6) addressing anti-vaccination rumors and increasing community acceptance of polio vaccines. All these factors were substantially significant in the milestone achieved by Nigeria and the certification of the WHO-AFR region in August 2020. It is highly recommended that the strategic sustenance of the EOC structures and polio assets post-certification of WPV interruption in Nigeria is crucial to sustaining polio eradication efforts for AFP disease surveillance and RI system strengthening. This is essential to address the challenge of stopping type 2 circulating vaccine-derived polioviruses outbreaks in Nigeria after the eradication of WPV in 2020. The documented lessons learned from this research study in Nigeria is a public health legacy of polio eradication in Africa for other vaccine-preventable disease programs, especially in the context of other African countries or the global south.

**Disclaimer:** the findings and conclusions in this report are those of the authors and do not necessarily represent the official position of the U.S. Centers for Disease Control and Prevention.

### What is known about this topic

- Towards the end of the 2010s decade, Nigeria, Afghanistan, and Pakistan were the only three countries globally where polio remained endemic;
- Nigeria experienced a setback with the detection of wild poliovirus (WPV) transmission in 2016 after going undetected for 2 years due to insecurity in the North-East;
- Nigeria eventually interrupted the transmission of WPV, and the World Health Organization Region of Africa was certified free of WPV as of August 2020.

### What this study adds

- A holistic review of the interruption of WPV transmission in Nigeria by the National Polio Emergency Operations Center that coordinates all Nigerian polio eradication efforts;
- The chronological events that impacted the epidemiology of WPV in Nigeria during 2000-2020 and the innovative strategies deployed to overcome the programmatic challenges encountered that contributed to flattening the WPV epidemic curve.
